# Robust Line-Scan Image Registration via Disparity Estimation for Train Fault Diagnosis

**DOI:** 10.3390/s25237315

**Published:** 2025-12-01

**Authors:** Darui Feng, Kai Yang, Zhi Ling, Yong Wang, Lin Luo

**Affiliations:** School of Physical Science and Technology, Southwest Jiaotong University, Chengdu 610031, China; dary_von@my.swjtu.edu.cn (D.F.); ling_zhizzz@126.com (Z.L.); wangyonga@swjtu.edu.cn (Y.W.); happyluolin@home.swjtu.edu.cn (L.L.)

**Keywords:** line-scan image registration, feature aggregation, high-speed railway, simulation dataset, fault diagnosis

## Abstract

Automatic fault detection based on machine vision technology is crucial for the operational safety of trains. However, when imaging moving trains, system errors may induce localized geometric distortions in the captured images, altering the shapes of critical train components. This, in turn, undermines the precision of subsequent diagnostic algorithms. Therefore, image registration prior to anomaly detection is essential. To address this need, we redefine the horizontal registration of line-scan images as a disparity estimation problem on rectified stereo pairs, which is solved using a proposed dense matching network. The disparity is iteratively refined through a GRU-based update module that constructs a multi-scale cost volume with positional encoding and self-attention. To overcome the absence of real-world disparity ground truth, we generate a physics-based simulation dataset by analytically modeling the nonlinear relationship between train velocity variations and line-scan image distortions. Extensive experiments on diverse real-world train image datasets under varied operational conditions demonstrate that our method consistently outperforms alternatives, achieving 5.8% higher registration accuracy and a fourfold increase in processing speed over state-of-the-art approaches. This advantage is particularly evident in challenging scenarios involving repetitive patterns or texture-less regions.

## 1. Introduction

In recent years, high-speed railway and urban metro networks have become indispensable modes of public transportation. As both ridership and route coverage have expanded markedly [1], operational safety and reliability requirements have intensified correspondingly. Consequently, systematic condition monitoring and fault diagnosis of key train components are now essential for ensuring uninterrupted service. Machine-vision-based diagnostic methods offer significant gains in inspection efficiency, accuracy, and scalability to address these challenges. However, a critical assumption underpinning many existing train inspection systems [2,3] is that the train’s velocity is perfectly matched with the line-scan camera’s acquisition rate. While real-time adjustment of the acquisition rate can mitigate this mismatch, residual distortions or misalignments invariably arise from systematic errors in the velocity measurement apparatus [4,5]. Applying diagnostic algorithms to such distorted images can easily generate false alarms. This underscores the necessity for accurate image registration as a prerequisite for reliable fault detection.

**Figure 1:**
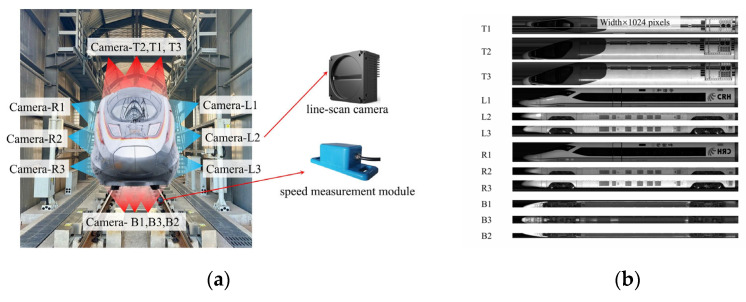
Train carriage image acquisition system. (**a**) Line-scan imaging equipment installed around the train inspection portal, equipped with a speed-measurement module that dynamically adjusts acquisition line rate (the letters T, L, R, and B denote the top, left side, right side, and bottom, respectively). (**b**) Carriage images captured from 12 cameras.

Train-inspection systems typically employ either area-array cameras or line-scan cameras. These systems integrate a variety of component technologies, such as stereo matching [6,7], point-cloud processing [8,9,10], 3D shape measurement [11,12,13], and high-precision image-registration-based anomaly detection [14,15]. Although area-array cameras can capture an entire image in single exposure, their limited field of view and the extreme aspect ratio of train carriage necessitate post-capture stitching of multiple sub-images to obtain a complete image. Variations in illumination and lens-edge distortion frequently impede precise alignment between adjacent images [16], posing substantial stitching challenges. By contrast, line-scan cameras acquire imagery single column at a time and exhibit minimal edge distortion, rendering them particularly well suited for high-resolution imaging of elongated subjects such as trains [17,18,19]. Figure 1a illustrates a representative line-scan carriage-imaging system capable of acquiring 360° views of passing trains for comprehensive exterior inspection. Registering two line-scan images of a train faces several significant challenges. First, the imagery includes both highly repetitive textures (e.g., grilles) and nearly texture-less regions (e.g., roof panels and side skirts). Dust accumulation further alters local appearance. These factors impair robust keypoint detection and increase the likelihood of mismatches. Second, spatially varying illumination and occasional specular reflections from metallic surfaces introduce additional appearance variations. This variability complicates descriptor matching. Finally, line-scan images are extremely large. Their heights are typically fixed at 1024 pixels, while their widths in our dataset range from 8192 to more than 32,760 pixels. Such large image sizes impose heavy computational and memory demands on registration algorithms.

Image registration is the process of aligning a pair of images via an appropriate transformation. Zitová and Flusser [20] categorized classical image registration techniques into two broad classes: intensity-based and feature-based methods.

As a specialized subset, existing registration approaches for line-scan images can likewise be divided into two types: intensity-based methods [21] and feature-based methods [1,22,23,24,25]. Intensity-based methods typically rely on template matching for alignment. For example, Song et al. [21] integrate classical template matching with the Enhanced Correlation Coefficient (ECC) algorithm. However, because these methods depend solely on the intensity of information, they often become stuck in local optima when applied to repetitive train-surface textures.

Feature-based methods are more widely used in practice. The time-scale normalization (TSN) algorithm by Lu et al. [22] aligns line-scan images by extracting SIFT [26] keypoints and estimating multiple affine transforms. Yet TSN accuracy degrades in low-texture regions where features are sparse, and the use of separate affine blocks can introduce cumulative misalignment at block boundaries. Chang et al. [23] address this feature scarcity by proposing Omnidirectional Scale Correlation Normalization (OSCN), which refines both keypoint detection and matching. Despite these enhancements, OSCN still fails in highly repetitive regions, leading to local registration breakdowns. Liu et al. [24] partition the full-train image into sub-blocks based on vehicle-body markers and optimize the stretch ratio of each block using mean-squared error (MSE). Although effective when such markers are present, this method incurs substantial computational overhead when processing large line-scan images, which undermines its real-time performance.

Deep-learning approaches have recently emerged to overcome these limitations. Fu et al. [1] apply a SuperPoint [27] sliding-window feature extractor, enforce geometric consistency via RANSAC [28] and a cubic-polynomial filter, and finally fuse correspondences using a weighted radial basis-function (WRBF) interpolation to register entire line-scan images. Although this pipeline accelerates overall alignment, its geometric parameters must be retuned for diverse locomotive types. Chang et al. [25] further improved robustness by encoding multi-scale features with a VGG-style backbone [29] and matching them with SuperGlue [30], then fitting a quintic polynomial to correct horizontal distortions. While this method achieves high accuracy, the quintic-fitting step incurs substantial computational cost, resulting in long per-image processing times. Feature-based registration methods remain the mainstream. However, in regions with repetitive patterns, sparse feature matching often leads to a high rate of incorrect correspondences, significantly degrading registration accuracy.

All of the aforementioned methods adopt a traditional multi-stage, pipeline-style architecture. Even when deep-learning modules are integrated into each stage, it remains difficult to achieve real-time performance. Critically, these existing algorithms derive registration transformations through multiple independent processing steps (e.g., feature extraction, matching, and transformation estimation). This multi-stage structure inevitably leads to the accumulation of errors, further limiting overall registration effectiveness.

Through our analysis of existing line-scan image registration methods, we recognize that, owing to the intrinsic imaging mechanism of line-scan cameras, horizontal alignment must be performed on entire pixel columns (hereafter referred to as line-pixels) rather than on individual pixels. Consequently, the primary challenge lies in estimating the shift of each line-pixel. Thus, we reformulate the problem as estimating line-disparity (defined as the horizontal offset between corresponding line-pixels) on epipolar-rectified stereo pairs, analogous to stereo disparity but specific to line-scan data. To train and evaluate our approach, we also introduce a physics-informed framework for synthesizing labeled line-scan image datasets directly from vehicle velocity profiles. Our key contributions are:

1. Mathematical modeling of line-scan imaging. We equate the line-scan camera’s acquisition rate to a velocity and derive a closed-form model that links train-velocity fluctuations to the resulting compressive and tensile deformations in line-scan images.
2. Simulation dataset generation. We introduce a radial basis function (RBF) method for synthesizing a labeled line-scan image dataset suitable for network training.
3. Reformulation line-scan image registration framework and performance breakthrough. By casting horizontal registration as a line-disparity estimation task and designing an efficient cost volume, our method substantially outperforms existing algorithms in both accuracy and speed, particularly in regions with weak or repetitive textures, while maintaining low memory consumption. To ensure that the estimated disparities and their induced transformations adhere to realistic train-motion characteristics, we further introduce a dual-smoothness loss function.

The remainder of this paper is organized as follows. Section 2 presents an overview of the proposed method. Section 3 details the construction of the simulated line-scan image dataset. Section 4 describes the line-disparity estimation-based registration algorithm. Section 5 reports experimental results, and Section 6 concludes the paper.

## 2. Methodology Overview

**Figure 2:**
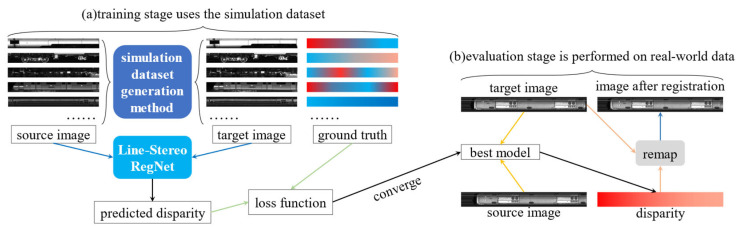
Overview of the proposed line-scan image registration method. We train our model only on simulation datasets and evaluate on test images captured from line-scan camera imaging system.

As illustrated in Figure 2a, our methodology begins with a physics-informed simulation process that generates training data with dense ground-truth line-disparity labels. Synthetic data are required because real train-inspection systems cannot measure pixel-level displacement during acquisition, making supervised learning otherwise infeasible.

A key concept of our framework is line-disparity, defined as the scalar horizontal offset shared by all pixels within a captured column. Since line-scan distortions are inherently one-dimensional, the registration problem can be reformulated as estimating this per-column displacement field, which corresponds mathematically to the integral of velocity fluctuations (Section 3).

The core of our approach is Line-Stereo RegNet, a compact architecture designed to predict the complete registration transformation in a single step, unlike existing multi-stage pipelines. The network is tailored to handle the extreme aspect ratios and repetitive textures of line-scan imagery. Given a source–target pair, it outputs a dense line-disparity map (Figure 2b), which is upsampled to full resolution and converted into a pixel-wise mapping function. The target image is finally warped via bilinear interpolation to obtain the geometrically corrected result.

## 3. Simulation Dataset Generation Method

One significant challenge in training a network to predict accurate line-pixel disparities is the absence of line scan image datasets annotated with ground truth line-disparity. In practice, two images of the same scene captured at different times serve as the source and the target. Any difference in velocity between these acquisitions appears as a positional shift when integrated over time. Specifically, if the source image is recorded at the reference velocity $v_{src}$, which ideally matches the camera acquisition line rate, then any deviation in the target velocity

(1) $$\Delta v(\tau )=v_{tar}(\tau )-v_{src}(\tau ),$$

when integrated over time, induces line-pixel displacements. Consequently, obtaining the ground truth line-disparity, that is the time integrated velocity difference between target and source captures, is essential for aligning the target image to the source image.

### 3.1. Mathematical Model of Line-Scan Camera Imaging Distortion

**Figure 3:**
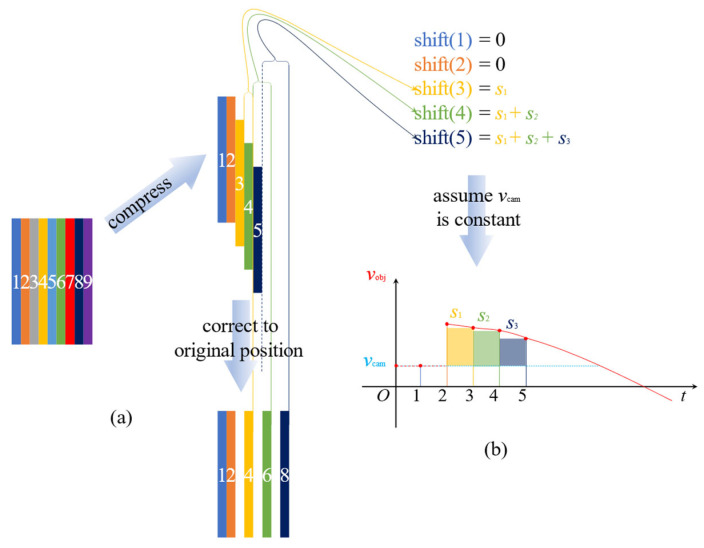
Distortion model illustrating compression effects in line-scan camera imaging. (**a**) Image registration corrects geometric distortion by realigning line-pixels to their original positions. (**b**) The shift distance of each line-pixel accumulates progressively along the imaging direction, with the magnitude dependent on its position in the image sequence.

Let the line acquisition rate be denoted as $f_{line}$ (in lines per second, Hz), and let $R_{y}$ represent the spatial resolution along the motion direction, defined as the physical dimension of a single pixel projected onto the object’s surface (e.g., meters per pixel). The camera’s equivalent scanning velocity $V_{cam}$ is then given by:

(2) $$V_{cam}=f_{line}\cdot R_{y},$$

where $f_{line}$ is the line acquisition rate and $R_{y}$ is the spatial resolution. Introducing the object velocity $V_{obj}$ (i.e., train velocity) enables a formal description of distortion in line-scan imaging systems. When $V_{obj}=V_{cam}$, the system operates in perfect synchronization: during each acquisition interval $1/f_{line}$, the train advances exactly one pixel length $R_{y}$. Each acquired line then corresponds to a unique, contiguous spatial segment of the object, yielding a geometrically accurate image. When $V_{obj}>V_{cam}$ (Figure 3a), the train’s velocity exceeds the camera’s equivalent scanning velocity. Consequently, within one acquisition interval, the train moves farther than $R_{y}$, causing spatial information loss between consecutive line acquisitions. This results in image compression along the motion axis as a larger physical length is mapped onto fewer line-pixels. A similar analysis applies when $V_{obj}<V_{cam}$, resulting in stretching rather than compression.

To quantify the distortion induced by non-uniform object velocity, we model the positional deviation of each line-pixel in the acquired image. Assume image acquisition begins at time *t* = 0, for simplicity, we first let $f_{line}=1\mathrm{Hz}$, such that the *n*-th line-pixel is captured at time

(3) $$t=\frac{n}{f_{line}}.$$

Let the instantaneous velocity of the train (object) be defined as a time-variant function $V_{obj}(t)$, and the camera’s equivalent scanning velocity as $V_{cam}(t)$. The actual physical distance traveled by the object from *t* = 0 to the capture of the *n*-th line is:

(4) $$y(t)=\int_{0}^{t}V_{obj}(\tau )d\tau ,$$

where y(t) corresponds to the true, corrected position of the *n*-th line-pixel, denoted as $p_{corr}(n)$. This is obtained by normalizing the physical distance by the spatial resolution $R_{y}$:

(5) $$p_{corr}(n)=\frac{y(t)}{R_{y}}=\frac{1}{R_{y}}\int_{0}^{t}V_{obj}(\tau )d\tau =\int_{0}^{t}v_{obj}(\tau )d\tau ,$$

where $v_{obj}(t)$ is the object velocity normalized to image space (i.e., in pixels per second). In the distorted image, the *n*-th captured line-pixel is located at the observed pixel position

(6) $$p_{obs}(n)=n.$$

The correct process as shown in Figure 3a, for the *n*-th line-pixel’s positional deviation, shift(n) is the difference between its true corrected position and its observed position in the distorted image:

(7) $$shift(n)=p_{corr}(n)-p_{obs}(n)=p_{corr}(n)-n.$$

To express n in terms of the acquisition velocity, we rewrite it as:

(8) $$n=\int_{0}^{t}v_{cam}(\tau )d\tau .$$

Substituting (4) and (6) into (5), we derive the final expression for distortion as the cumulative difference in velocities (see Figure 3b):

(9) $$shift(n)=\int_{0}^{t}[v_{obj}(\tau )-v_{cam}(\tau )]d\tau .$$

In our proposed framework, this shift is interpreted as line-disparity S(n). Assuming the source image is acquired under constant velocity $v_{src}=v_{cam}$, the target disparity becomes:

(10) $$S(n)=\int_{0}^{t}[v_{tar}(\tau )-v_{src}(\tau )]d\tau .$$

### 3.2. Generation of Velocity Profiles

Accurate velocity profiles are critical; datasets such as FlyingChairs [31] rely on simple affine transformations, which cannot capture the random compressive/stretching distortions characteristic of train line-scan imagery, and thus fail to ensure network robustness. Drawing on studies in train speed-control systems [32,33], and according to (2), where each line-pixel position can be equated to *t*, we model the instantaneous velocity of the train within the camera’s field of view using a radial basis function (RBF) expansion:

(11) $$v(t)=\sum_{i=0}^{N-1}{\hat{w}}_{i}\cdot \phi (t,c_{i}),c_{i}(i=0,1,\cdots ,N-1),$$

where $c_{i}$ are control point locations, *N* is the number of control points, and $\phi (t,c_{i})$ is Gaussian kernel

(12) $$\phi (t,c_{i})=\exp\left(-\frac{{\left(t-c_{i}\right)}^{2}}{2\sigma^{2}}\right).$$

The weights ${\hat{w}}_{i}$ are drawn from a normal distribution, and then normalized:

(13) $$w_{i}\sim N(0,1),\;{\hat{w}}_{i}=\frac{w_{i}}{\sum_{j=0}^{N-1}|w_{j}|}$$

where j=0,1,2,⋯,N−1, $w_{i}$ are weights from original normal distribution. To facilitate analytical integration, we fit the discrete RBF samples v(t) with a cubic spline, yielding a continuous velocity curve $v_{tar}\left(t\right)$. To mimic the carriage–segmentation errors observed in practice, we introduce an initial offset $\delta_{0}$. In our implementation, we set $v_{\mathrm{src}}\left(t\right)=1$ pixel/s, the resulting disparity becomes:

(14) $$S(n)=\delta_{0}+\int_{0}^{t}\left[v_{tar}\left(\tau \right)-1\right]d\tau .$$

### 3.3. Augmentation Strategy

**Figure 4:**
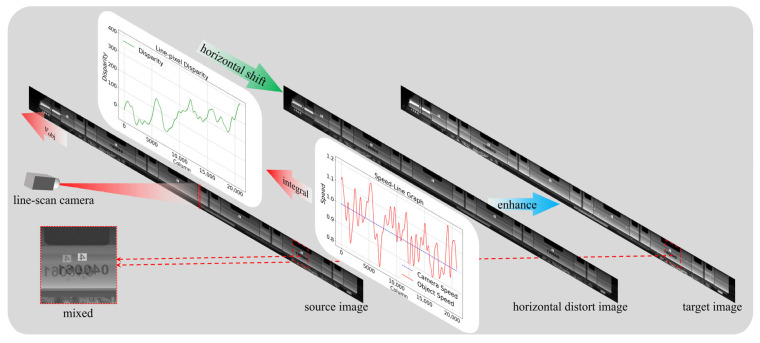
Workflow for generating the simulation line-scan image registration dataset.

Real-world disturbances such as camera vibration, exposure fluctuations and random reflections from metal surfaces induce multidimensional differences between historical and current frames. As shown in Figure 4, after applying the horizontal disparity S(n) to the source image, we obtain an intermediate, horizontally warped image $I_{\mathrm{hor}}$. We then define the augmented target image $I_{\mathrm{en}}$ as:

(15) $$I_{\mathrm{en}}=G(R(I_{\mathrm{hor}},Y)),$$

where Y is a vertically periodic shift field

(16) $$Y=A\cdot \cos\left(\frac{2\pi }{T}\cdot x+\phi_{0}\right),$$

where T,A,G and R are period, amplitude, initial phase, random exposure-gain function and specular-highlight noise, respectively. This composite procedure yields realistic target images for robust network training.

Our final simulation dataset is illustrated in Figure 4. Briefly, we first compute the line-disparity for the source image (whose velocity profile is shown by the red curve in the Speed-Line Graph). We then integrate the velocity profile over time to obtain the line- disparity (green curve in the Line-pixel Disparity graph) and apply this line-disparity to generate a horizontally distorted version of the source image. Additional augmentations are introduced to produce the target image. Finally, we crop identical regions from the source and target images to create a composite. This composite exhibits horizontal and vertical offsets that faithfully replicate the true distortion patterns found in train line-scan data.

### 3.4. Simulation Fidelity and Implementation Assumptions

Our simulation framework links synthetic labels to real data by modeling the physical cause of line-scan distortions, namely velocity asynchrony. Instead of simple affine augmentations, it analytically derives the nonlinear line-disparity field from train velocity profiles and combines these horizontal distortions with vertical periodic shifts as well as illumination and noise variations. This produces deformations that closely match real inspections and enables the network to learn geometric behavior from physical principles rather than surface appearance.

The simulation is based on standard operational assumptions. We focus on geometric distortion and assume high optical quality, which aligns with modern systems that employ strobe lighting and synchronized shutters. As a result, degradations such as blur or exposure lag are not modeled, since they seldom occur in properly calibrated setups. This abstraction allows the network to concentrate on the primary challenge, namely strong nonlinear geometric distortions in repetitive or low-texture regions, while extreme failures such as severe motion blur remain outside the scope of this task.

## 4. Disparity Estimation Based Line-Scan Image Registration Network

To predict the line-disparity between source and target line-scan image, and inspired by the optical flow estimation network Flow1D [34] and RAFT [35], we propose Line-Stereo RegNet, which introduce a feature-aggregation scheme tailored to disparity estimation on line-scan images. This scheme not only strengthens the representational capacity of line-scan features but also minimizes computational overhead.

### 4.1. Feature Extraction and Reorganization

**Figure 5:**
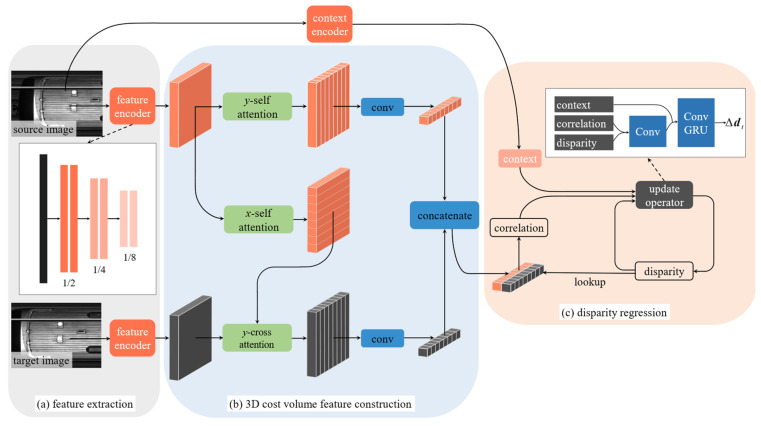
Network structure of proposed Line-Stereo RegNet. (**a**) Extract feature by residual blocks. (**b**) Construct 3D cost volume with attention mechanism. (**c**) GRU-based disparity regression scheme.

As illustrated in Figure 5a, our feature-extraction backbone consists of six residual blocks that output feature maps at one-eighth of the input resolution. To help infer occluded regions and prevent disparity misestimation, we also extract contextual features from the source image using an identical network. Given a pair of feature maps $F_{1}$, $F_{2}\in {\mathbb{R}}^{H\times W\times D}$ (where *H*, *W*, *D* denote height, width, and feature dimension, respectively), our goal is to produce a reorganized feature $F_{1,y}$ in which each feature vector at (*h*, *w*) incorporates information from all features in the same column. This facilitates one-dimensional horizontal search after collapsing the vertical dimension.

To meet the challenges posed by extensive low-texture and repetitive regions in train imagery, we draw on the success of Transformer-based global contextual information in weak-texture matching (e.g., LoFTR [36]). We first add a shared positional encoding for $F_{1}$ and $F_{2}$, to get ${\tilde{F}}_{1}=F_{1}+P$, ${\tilde{F}}_{2}=F_{2}+P$. To enable each spatial location in the feature map $F_{1}$ vertically reorganized, we apply a y-self attention mechanism. This process aims to refine the features, making them more robust to occlusions and repetitive textures. Specifically, the Query, Key, and Value are all derived from $F_{1}$:

(17) $$\begin{array}{l} Q_{1}=W_{Q1}({\tilde{F}}_{1})\\ K_{1}=W_{K1}({\tilde{F}}_{1})\\ V_{1}={\tilde{F}}_{1}, \end{array}$$

where $W_{Q1}$ and $W_{K1}$ are 1 × 1 convolutional layers serving as linear transformations. The attention is computed independently for each horizontal position (i.e., for each column of width W by permuting the tensor dimensions to allow attention to operate along the vertical height H) dimension. The resulting reorganized feature $F_{1,y}$ is expressed as:

(18) $$F_{1,y}=\mathrm{softmax}(\frac{Q_{1}K_{1}^{T}}{\sqrt{C}})V_{1},$$

where C is a scaling factor that prevents extreme values.

An analogous procedure produces the horizontal reorganized $F_{1,x}\in {\mathbb{R}}^{H\times W\times D}$. Subsequently, we use $F_{1,x}$ as the source for the query and perform y-cross attention on the feature map $F_{2}$. This allows the context-aware features at each position in $F_{1}$ to find the most relevant correspondences along the vertical axis of $F_{2}$. The Query, Key, and Value are defined as:

(19) $$\begin{array}{l} Q_{2}=W_{Q2}(F_{1,x})\\ K_{2}=W_{K2}({\tilde{F}}_{2})\\ V_{2}={\tilde{F}}_{2}, \end{array}$$

where $W_{Q2}$ and $W_{K2}$ are independent 1 × 1 convolutional layers. Similar to the previous step, this attention operation is also performed along the vertical dimension. The resulting reorganized feature $F_{2,y}$ is then given by:

(20) $$F_{2,y}=\mathrm{softmax}(\frac{Q_{2}K_{2}^{T}}{\sqrt{C}})V_{2}.$$

Thus, $F_{1,y}$ integrates the intra-image vertical context of $F_{1}$, which is crucial for stabilizing matching in texture-sparse or highly repetitive regions, thereby significantly improving the final matching accuracy; while $F_{2,y}$ represents a vertically reorganized representation of $F_{2}$, guided by the horizontal context of $F_{1}$. This vertical reorganization operation, tailored for the 1D distortion characteristics of line-scan imaging, substantially reduces the dimensionality of the cost volume computation, thus significantly boosting computational efficiency and memory utilization. These enhanced features establish a robust foundation for constructing a more effective cost volume (see Figure 5b).

### 4.2. Cost-Volume Construction via One-Dimensional Correlation

After reorganizing the features, we construct the cost volume based on standard stereo matching principles. Since all pixels within a single column of a line-scan image are captured simultaneously, retaining full vertical information is unnecessary. Moreover, the large size of line-scan images imposes prohibitive memory requirements. Inspired by TSN’s aggregation approach, we apply an H×1 convolution to $F_{1,y}$ and $F_{2,y}$ to collapse their height dimension, yielding the aggregated features ${\hat{F}}_{1}$, ${\hat{F}}_{2}\in {\mathbb{R}}^{1\times W\times D}$. We then perform a horizontal search with radius *R* to construct a cost volume $C\in {\mathbb{R}}^{1\times W\times (2R+1)}$:

(21) $$C(1,w,R+r)=\frac{1}{\sqrt{D}}{\hat{F}}_{1}(1,w)\;\cdot {\hat{F}}_{2}(1,w+r)$$

where r∈{−R,−R+1,⋯,0,⋯,R−1,R}, and · denotes the inner product and $1/\sqrt{D}$ normalizes the result, respectively. In practice, we first compute a full 1×W×W matrix of inner products between ${\hat{F}}_{1}$ and ${\hat{F}}_{2}$ along the width dimension, and then extract the band of width 2R+1 around the diagonal via sliding-window indexing, thereby efficiently realizing the above expression. Thanks to attention-enhanced features, this simple one-dimensional search suffices to match line-pixels between source and target.

### 4.3. Disparity-Regression Framework

As shown in Figure 5c, we adopt an iterative scheme to refine the disparity estimate. Given the constructed cost volume $C\in {\mathbb{R}}^{1\times W\times (2R+1)}$, at each iteration t, an update operator predicts a disparity increment $\Delta d_{t}$. Specifically, the current disparity estimate $d_{t-1}$ is used to index into C and extract a correlation feature map; concurrently, a context feature encoder which shares the same architecture as the backbone extracts contextual cues from the source image. These three inputs (correlation features, current disparity estimate, and context features) are fed into the update operator: after convolving the indexed cost volume and $d_{t-1}$, their outputs are merged with the context features and passed through a Gated Recurrent Unit (GRU), which yields $\Delta d_{t}$. The disparity is then updated by $d_{t}=d_{t-1}+\Delta d_{t}$. In our implementation, we perform *N* = 12 iterations. The lookup operation is defined such that, for a pixel at (1,w) with estimated disparity $d=f_{x}$, the center of the search window is at $\left(1,w+f_{x}\right)$ and the cost volume within radius R is indexed for regression.

The refinement stage is essential for converting the coarse disparity map into a highly accurate result. We adopt a GRU-based update module for two key reasons. First, its recurrent structure enables iterative error correction, allowing the model to capture nonlinear distortions and achieve superior sub-pixel accuracy beyond single-pass methods. Second, the GRU is lightweight and parameter-efficient, offering a much cheaper alternative to deep convolutional refinement networks. This compact design keeps refinement overhead minimal and supports the real-time performance of Line-Stereo RegNet.

### 4.4. Loss Functions

For horizontal registration, we first define a sequence loss over the predicted disparity $\left\{d_{1},\ldots ,d_{N}\right\}$ and the ground truth $d_{\mathrm{gt}}$. We weight each iteration’s $l_{1}$ error by an exponentially decaying factor $\gamma^{N-i}$:

(22) $$L_{\mathrm{series}}=\sum_{i=1}^{N}\gamma^{N-i}\|d_{\mathrm{gt}}-d_{i}{\|}_{1}$$

where γ=0.8, N=12 in our implementation. To remap the target image, we define the horizontal coordinate mapping:

(23) m(x)=x+d(x).

Let x denote the line-pixel coordinate in the target image and d(x) its estimated disparity. Given that trains exhibit smooth velocity transitions, we introduce a dual smoothness loss to enforce regularity in both the disparity field d(x) and the mapping m(x) to be smooth. This regularization term combines first- and second-order derivative penalties on d(x) and m(x):

(24) $$\begin{array}{l} L_{\mathrm{smooth}}=\lambda_{1}L_{\mathrm{disp}}^{\left(1\right)}+\lambda_{2}L_{\mathrm{disp}}^{\left(2\right)}+\lambda_{3}L_{\mathrm{map}}^{\left(1\right)}+\lambda_{4}L_{\mathrm{map}}^{\left(2\right)}\\ L_{\mathrm{disp}}^{\left(1\right)}=\frac{1}{W}{\left(\partial_{x}d(x)\right)}^{2},\;L_{\mathrm{disp}}^{\left(2\right)}=\frac{1}{W}{\left(\partial_{xx}d(x)\right)}^{2}\\ L_{\mathrm{map}}^{\left(1\right)}=\frac{1}{W}{\left(\partial_{x}m(x)\right)}^{2},\;L_{\mathrm{map}}^{\left(2\right)}=\frac{1}{W}{\left(\partial_{xx}m(x)\right)}^{2} \end{array}$$

where $\partial_{x}$ and $\partial_{xx}$ denote the first- and second-order derivatives along x, respectively, and $\lambda_{i}(i=1,2,3,4)$ are weighting coefficients. The total loss function integrates sequence and smoothness regularization:

(25) $$L_{\mathrm{total}}=L_{\mathrm{series}}+L_{\mathrm{smooth}}.$$

Finally, the registered image s generated by warping the source image via bilinear interpolation according to the mapping function ***m***(*x*), as illustrated in Figure 2.

## 5. Experiments and Evaluation

To thoroughly assess the performance of the proposed line-scan image registration algorithm (Line-Stereo RegNet), we perform both qualitative and quantitative evaluations across multiple vehicle-type datasets, comparing against current mainstream methods, including TSN, OSCN, and the method proposed in [1] (hereafter referred to as WRBF for clarity). Additionally, comprehensive ablation studies are conducted to validate the contributions of the feature reorganize module, then discuss the effectiveness of dual smoothness loss and the simulation dataset for our research. To ensure experimental consistency and reproducibility, all evaluations are carried out in a standardized hardware and software environment: an Intel i9-12900K CPU @ 3.9 GHz, an NVIDIA GeForce RTX 4090 GPU (24 GB VRAM), and 64 GB of RAM. The environment includes Windows 11, Python 3.8, CUDA 11.3, and PyTorch 1.10.0.

### 5.1. Dataset and Experiment Configuration

To evaluate the generalization capability of the proposed method, we collected 4569 real-world line-scan image pairs from nine installation positions across diverse high-speed rail (CR400BF, CRH380A, CRH2A, CR200) and subway trains (GZ18, SH6, WX4, NN4). Images were acquired under varying weather and illumination conditions at train velocities of 20–25 km/h (Figure 1). All images have a fixed height of 1024 pixels, with widths varying by train model and camera position, yielding aspect ratios of ~7:1 to 32:1. Using the method described in Section 3, we further generated a simulated dataset comprising 9138 image pairs based on the source images. This dataset is divided into training and validation subsets at an approximate ratio of 5:1, resulting in 7615 pairs for training and 1523 pairs for validation. The original 4569 real-collected image pairs are used as a test set. To accelerate convergence and improve final accuracy, we adopt a two-stage training strategy. In the pretraining stage, input images are downscaled to 256 × 256 pixels. Training employs a search radius of *R* = 64, an initial learning rate of 1.25 × 10^−4^ with a multiplicative decay factor of 10^−5^ per iteration, and a batch size of 4. After 10,000 steps of training, a coarse disparity-aware pretrained model is obtained. In the fine-tuning stage, images are resized to a high-resolution format of 256 rows × 16,384 pixels. The learning rate is reduced to 1.25 × 10^−5^, and the batch size is decreased to 2, while all other hyperparameters remain unchanged. The pretrained model initializes the network weights, and fine-tuning proceeds for an additional 160,000 iterations on the same dataset. Both stages utilize the Adam optimizer.

### 5.2. Ablation Study

This section presents an ablation study to validate the efficacy of the primary components within our proposed matching cost volume construction methodology. The study systematically evaluates the contributions of self-attention, cross-attention, and positional encoding, following the experimental protocols detailed in Section 5.

The quantitative results of this study, as shown in Table 1, first underscore the fundamental importance of the attention framework. When both self-attention and cross-attention mechanisms were omitted, the model’s performance decreased substantially, with the mean SSIM score dropping from 0.8812 to 0.6037. This degradation was accompanied by a marked increase in result variance, as indicated by the standard deviation. Concurrently, the average memory usage was reduced from 3.319 GB to 2.545 GB. These results confirm that the integrated attention mechanisms are critical for achieving high-quality registration, despite their computational cost. Moreover, they demonstrate that our algorithm’s overall memory footprint remains remarkably low even when processing ultra-large, high-aspect-ratio images, highlighting its strong practical applicability.

Further analysis of the individual components reveals that cross-attention is the most critical element for the matching task. Its removal resulted in the most significant performance decline among all single-component ablations, reducing the SSIM score to 0.8036. This finding indicates that the direct establishment of inter-image feature correspondences via cross-attention is the principal contributor to the model’s accuracy.

The roles of self-attention and positional encoding were also found to be significant. The exclusion of the self-attention module led to a notable reduction in performance, with the SSIM score decreasing to 0.8377 and memory usage slightly declining to 3.118 GB. This suggests that the self-attention mechanism effectively enhances feature representations by capturing long-range intra-image dependencies, which is particularly valuable for large-aspect-ratio line-scan imagery. Similarly, removing positional encoding caused a comparable performance drop, with the SSIM score falling to 0.8357. The “w/o pos” variant unexpectedly consumed slightly more memory than the full model (3.351 GB vs. 3.319 GB). This suggests that positional encoding provides a useful prior that guides attention toward relevant regions. Without this spatial guidance, attention becomes more diffuse during backpropagation, which may reduce the effectiveness of underlying framework optimizations and result in a small increase in memory usage. This demonstrates that positional information is crucial for providing the spatial priors necessary to disambiguate features, especially in regions with repetitive or weak textures.

In summary, the ablation study confirms that the superior performance of our method stems from the synergistic integration of its core components. The architecture effectively fuses local features with global context, where cross-attention serves as the primary matching engine, self-attention functions as a feature enhancement module and positional encoding supplies essential spatial constraints. This combination enables robust and precise registration of challenging line-scan images, characterized by textural ambiguities and large spatial extents, while operating within a defined computational budget. It also highlights our algorithm’s significant low-memory advantage on ultra-large image inputs, underscoring its excellent value for real-world deployment.

### 5.3. Ablation Study Comparative Experiments

To comprehensively assess the registration performance of various algorithms under different levels of texture complexity (texture-less, repetitive textures, and rich texture details), we systematically selected three representative line-scan camera positions from nine installation points across eight train models: T1 (train top), L3 (train left side), and B3 (train bottom). From these camera views, 50 pairs of real-world images were randomly sampled and used for the final registration performance comparison, with visual assessments of registration results for a comprehensive judgment.

As the source codes for the baseline methods (TSN, OSCN, and WRBF) are not publicly available, and our dataset differs from those used in previous works, we reimplemented these methods based on their source paper descriptions. The results reported below are the outputs of these reproductions evaluated on our test dataset.

#### 5.3.1. Qualitative Assessment of Image Registration

**Figure 6:**
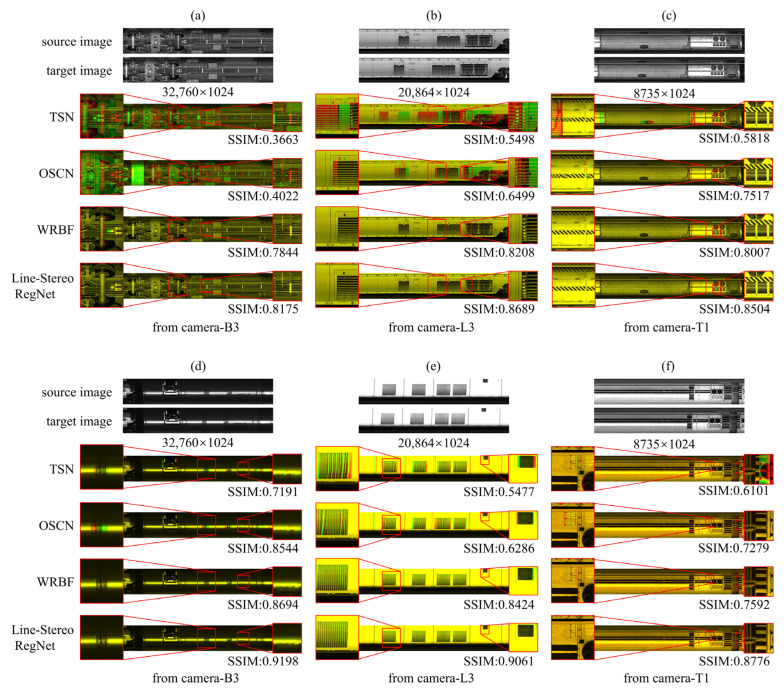

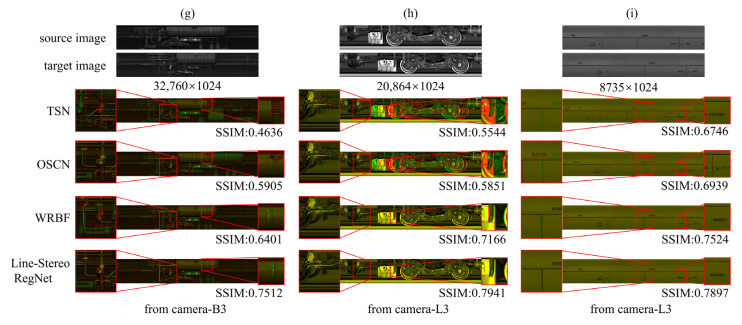
Qualitative comparison of registration results using different algorithms. Column 1: Middle-bottom camera image, characterized by rich texture facilitating feature matching. Column 2: Left-side camera image, presenting challenges including repetitive structures and low-texture regions. Column 3: Top and left-side camera image, dominated by low-texture content.

Figure 6 provides a qualitative comparison of Line-Stereo RegNet against three baselines (TSN, OSCN, WRBF) on real-world line-scan imagery. Registration accuracy is visualized by overlaying the registered red channel onto the reference green channel: yellow indicates proper alignment, while distinct red or green regions signify misalignment. Line-Stereo RegNet demonstrates robust and superior performance across most imaging conditions. In scenarios with severe nonlinear distortions (Column 1), conventional feature-point methods like TSN and OSCN exhibit significant limitations due to their reliance on piecewise affine transformations, leading to cumulative error and suboptimal solutions. Both WRBF and our method are better at modeling complex global nonlinear deformations, with Line-Stereo RegNet showing superior accuracy, particularly in wheelset regions.

Our Feature Reorganization Mechanism directly addresses distinct texture challenges. (1) For repetitive patterns (e.g., intake grilles, Column 2), *y*-axis self-attention aggregates global vertical context, capturing the unique vertical arrangement of a column. This disambiguates identical horizontal features (slats), preventing cycle-skipping and misalignment. (2) For large low-texture regions (e.g., smooth roof panels, Column 3), cross-attention functions as a context propagator, diffusing distinct features from boundaries into the textureless interior. This enables robust correspondence establishment even without prominent local keypoints. This general advantage is supported by integrated positional embeddings, which capture long-range dependencies for robust matching under significant displacement.

Despite its general superiority, the method exhibits limitations in extreme scenarios (Row 3, Figure 6). (1) Large disparity variations (Figure 6g,h): Registration fails when displacement exceeds 512 pixels. This boundary is a hard limit set by the network’s architecture (8× downsampling factor × 64 search radius). (2) Severe low-texture (Figure 6i): Matching ambiguity persists due to the absolute lack of discriminative features. It is notable, however, that even in these extreme cases, our approach preserves more structural details compared to existing methods, which are prone to catastrophic artifacts.

#### 5.3.2. Quantitative Assessment of Image Registration

Line-scan image registration for high-speed rail and subway systems faces several different challenges that call for separate evaluation. High-speed trains usually traverse at over 250 km/h, where strong vibrations, nonlinear geometric distortions, motion blur and rapidly shifting outdoor illumination severely degrade the registration algorithms’ effect, especially in texture-poor regions. By contrast, subway trains run through enclosed tunnels under steady artificial lighting, with minimal vibration and more varied, distinctive surface textures, which greatly reduces blur and lighting artifacts.

**Figure 7:**
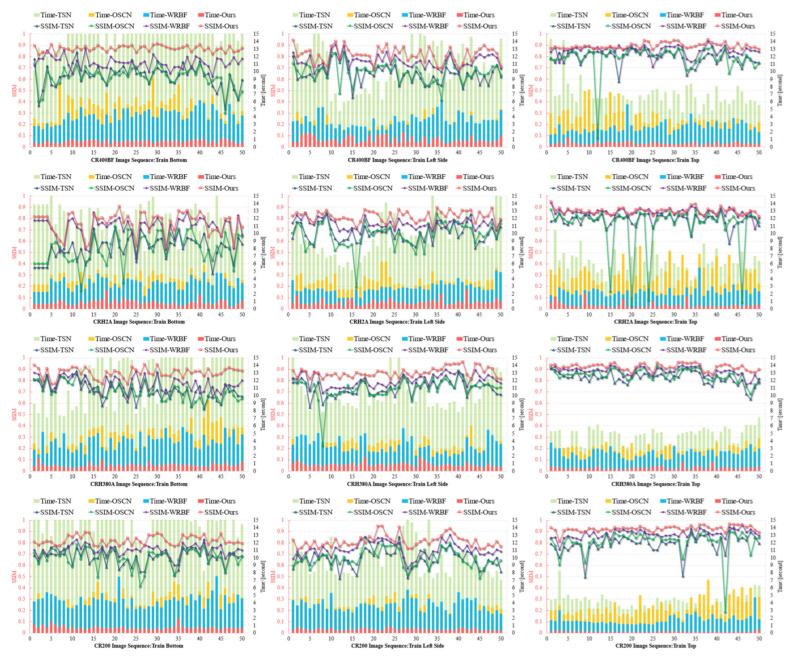
Comparison of registration accuracy and processing time on high-speed train images.

We evaluate our registration algorithms using two independent test sets. As illustrated in Figure 7, we used images captured at three fixed camera positions on four high-speed train models. In each subfigure, the horizontal axis represents 50 complete image pairs; the overlaid line and bar charts, distinguished by contrasting colours, depict the registration SSIM and per-pair processing time of each algorithm, respectively, with values referenced against the left and right vertical axes. The results show that TSN and OSCN demonstrated comparable registration accuracy metrics, though both exhibited fundamental limitations inherent to sparse feature-based matching paradigms. These methods consistently suffered from matching ambiguities in regions characterized by repetitive textural patterns (a prevalent condition in high-speed train exteriors, see train left side image sequence) due to their reliance on local feature point correspondences without adequate global context integration. WRBF achieved superior registration accuracy through its sliding window matching strategy, which effectively addressed some nonlinear deformation challenges. However, this approach incurred substantial computational overhead, particularly in texture-rich (train bottom image sequence) or ultra-wide format images, resulting in processing times greater than the proposed method. The proposed Line-Stereo RegNet achieved both higher precision metrics and significantly reduced processing latency. Statistical analysis revealed that Line-Stereo RegNet improves average registration error by 5.8% and the average processing time accounts for one-fourth relative compared to WRBF. These improvements demonstrate a theoretically and practically superior balance between registration fidelity and operational efficiency, particularly valuable for real-time high-speed rail inspection systems.

**Figure 8:**
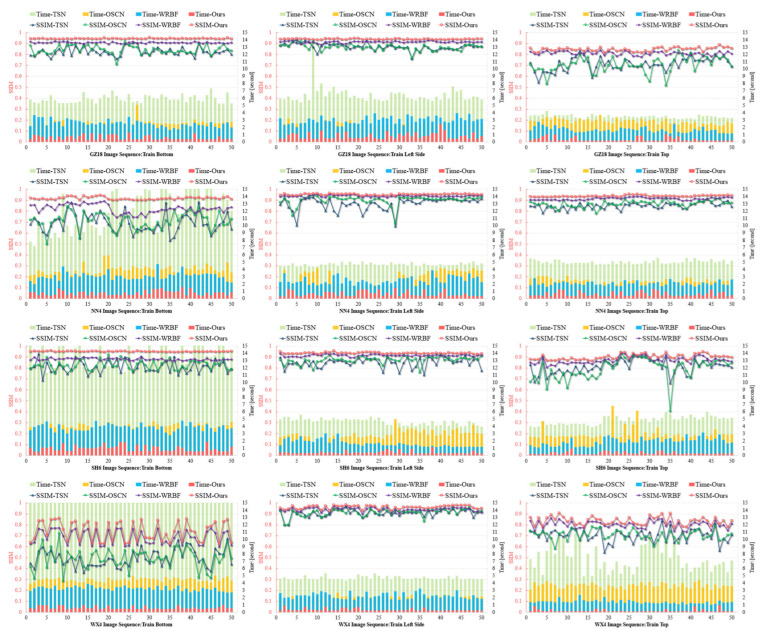
Comparison of registration accuracy and processing time on subway train images.

In subway train image experiments (see Figure 8), characterized by high grayscale consistency, minimal vibration interference and reduced illumination variation, the registration accuracy of traditional feature-based methods (TSN and OSCN) improved substantially. However, their computational performance remained constrained by the inherent overhead of multi-stage processing pipelines, including separate feature extraction, matching, and transformation estimation phases. WRBF further enhanced registration accuracy under these stable conditions through its refined spatial transformation model, though its computational efficiency remained suboptimal due to iterative refinement requirements. Line-Stereo RegNet outperforms other methods across almost all evaluation metrics, achieving higher registration accuracy. This advantage arises from its end-to-end architecture, which effectively eliminates error accumulation between traditionally separate processing stages. In low-texture, grayscale-uniform underground environments, Line-Stereo RegNet demonstrates exceptional robustness, maintaining precise alignment even on large homogeneous surfaces that typically challenge conventional approaches. In challenging train bottom scenes where many state- of-the-art pipelines experience a significant increase in processing time, our method adds only minimal computational overhead. This efficiency results from training on a highly diverse dataset, which enables rapid convergence and equips the model with the resilience to handle complex real-world situations with almost no extra runtime. The comprehensive experimental analysis across distinct operational environments and vehicle types confirms that the proposed algorithm achieves superior registration accuracy and computational performance across diverse imaging conditions. Its architectural advantages become particularly pronounced in the challenging high-speed rail scenarios, where it effectively addresses the combined challenges of repetitive textures, vibration-induced geometric distortions, and dynamic lighting variations through its integrated attention mechanisms and positional encoding framework.

### 5.4. Discussion

While Section 3 introduces a synthetic dataset generation workflow incorporating both horizontal shifts and vertical distortions, our network architecture remains specifically optimized for horizontal registration. To address this directional specialization, Section 4 proposes a dual smoothness loss that regularizes line-disparity predictions and transformation mappings. Consequently, this section examines them.

#### 5.4.1. Impact of Vertical Shift in Dataset on Registration Performance

While the primary focus of this research centers on horizontal registration challenges, the inclusion of vertical shifts in training data proves equally critical for developing robust real-world applications. To systematically evaluate this factor, we generated two synthetically distorted datasets following the methodology detailed in Section 3: one incorporating only horizontal displacements and another encompassing both horizontal and vertical distortions. Both datasets underwent identical training protocols and were evaluated under strictly controlled testing conditions to ensure a fair comparison. The quantitative results presented in Table 2 demonstrate that models trained on datasets incorporating vertical displacements achieve superior performance and better metrics, exhibiting higher average structural similarity indices and significantly reduced standard deviations compared to their horizontally constrained counterparts. This performance improvement indicates that explicit consideration of vertical geometric variations during training substantially enhances the model’s ability to handle real-world perturbations caused by factors such as camera height fluctuations and vehicle-induced vibrations. The enhanced robustness observed in these experiments underscores the importance of comprehensive data augmentation strategies that account for multi-directional geometric variations, ultimately contributing to improved generalization capabilities and operational reliability in practical deployment scenarios.

#### 5.4.2. Impact of Dual Smoothness Loss on Registration Performance

To isolate and quantify the impact of the dual-smoothness loss on registration accuracy, we fine-tuned two otherwise-identical models, one with and one without the dual-smoothness term, for 160,000 iterations from the same pretrained weights. Both models achieved nearly equivalent training sequence losses ($L_{\mathrm{series}}$ = 3.20 without dual-smoothness vs. $L_{\mathrm{series}}$ = 3.26 with dual-smoothness), ensuring that any observed differences in test-time performance arise from the regularizer itself rather than from disparities in convergence. As shown in Table 3, evaluation on the dataset described in Section 5.2 reveals that incorporating dual smoothness yields a significant increase in mean SSIM and a marked reduction in inter-sample performance variance. This improvement is attributable to the loss’s simultaneous enforcement of spatial coherence in both the disparity and optical-flow domains: it suppresses spurious local fluctuations while preserving salient structural boundaries. The dual regularization is especially beneficial in regions that traditionally challenge registration—namely, repetitive-texture areas prone to ambiguous matches and low-texture zones lacking distinctive features. By balancing the competing objectives of geometric fidelity and boundary preservation, the dual-smoothness loss not only elevates overall registration accuracy but also enhances robustness across a diverse array of imaging scenarios.

**Figure 9:**
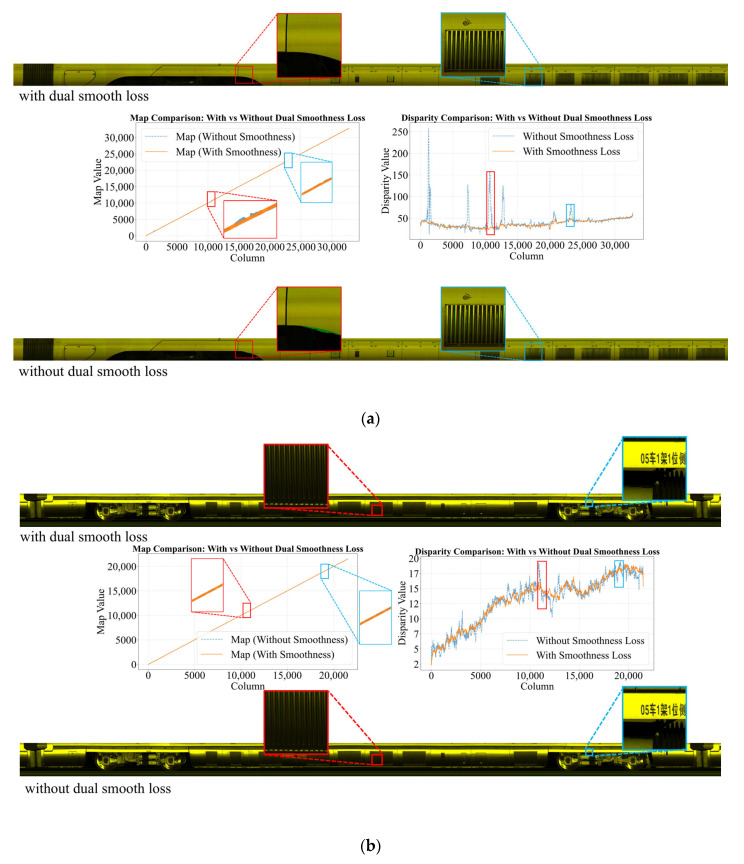
Impact of dual smoothness loss on Camera-L3 registration performance. (**a**) Without regularization, minor curvature in the mapping function amplifies into significant disparity distortion, inducing registration error. (**b**) Despite a normal mapping function, unregularized disparity estimation remains erroneous, demonstrating sensitivity to smoothness constraints.

Further qualitative analysis (Figure 9) highlights the crucial role of the dual smoothness loss and the complementary interaction between its first- and second-order terms. The second-order term maintains motion continuity and prevents disparity jumps in repetitive-texture regions, while the first-order term suppresses noise and avoids global drift in low-texture areas [37]. Together, they preserve structural consistency and numerical stability, enabling our framework to achieve state-of-the-art performance.

## 6. Conclusions

This study presents a robust framework for line-scan image registration that effectively addresses geometric distortions induced by velocity fluctuations between moving trains and line-scan cameras. By reformulating the horizontal registration problem as a line-disparity estimation task on epipolar-rectified stereo pairs, we develop an end-to-end deep learning network that seamlessly integrates physics-based simulation, attention-driven feature reorganization, and iterative disparity refinement. Our approach overcomes critical challenges in repetitive and texture-deficient regions through global contextual modeling while efficiently processing ultra-large line-scan images (up to 32,760 pixels wide) with remarkably low memory consumption (approximately 3.3 GB). Comprehensive validation across diverse real-world datasets from eight train models demonstrates that our method consistently outperforms state-of-the-art techniques, improving registration accuracy by 5.8% while requiring only one-fourth of the processing time on average. The integration of velocity-profile-based synthetic data generation and dual-smoothness regularization significantly enhances robustness against real-world perturbations, including camera vibrations and illumination variations. This work establishes a scalable solution for high-precision train inspection systems, with demonstrated applicability to other line-scan imaging domains requiring geometric fidelity under dynamic motion conditions. The framework’s balance of accuracy, efficiency, and practical deployability represents a significant advancement toward reliable automated fault diagnosis in transportation infrastructure inspection.

Looking ahead, our future work will focus on three main directions. First, we will further advance 2D high-precision registration by exploring cascaded architectures and extending the framework to multi-camera fusion. Second, we plan to enhance robustness to extreme motion-induced distortions through hierarchical coarse-to-fine models capable of handling larger disparity ranges. Finally, we aim to optimize the network for real-time deployment on embedded edge devices and broaden the applicability of our framework to a wider range of high-speed line-scan inspection scenarios.

## Figures and Tables

**Table 1:** Ablation Study on Cost Volume Construction Methods (✓ denotes enabled, ✗ denotes disabled).

Method	Self	Cross	Pos	Memory Usage	SSIM (Mean ± SD)
w/o self & cross	✗	✗	✓	**2.545 GB**	0.6037 ± 0.1334
w/o self	✗	✓	✓	3.118 GB	0.8377 ± 0.1206
w/o cross	✓	✗	✓	2.998 GB	0.8036 ± 0.1125
w/o pos	✓	✓	✗	3.351 GB	0.8357 ± 0.0791
Line-Stereo RegNet	✓	✓	✓	3.319 GB	**0.8812 ± 0.0734**

**Table 2:** Study on the Necessity of Vertical Shift in Dataset Construction.

Method	SSIM (Mean ± SD)
without vertical shift	0.8354 ± 0.1102
with vertical shift	0.8812 ± 0.0734

**Table 3:** Average SSIM Results of Study on Dual Smoothness Loss.

Method	SSIM (Mean ± SD)
without dual smooth loss	0.8275 ± 0.1189
with dual smooth loss	0.8812 ± 0.0734

## Data Availability

The generated data supporting the findings are currently not publicly accessible, but may be obtained from the authors upon reasonable request.
